# Impact of a Public Health Awareness Campaign on Patients’ Perceptions of Expanded Pharmacy Services in South Dakota Using the Theory of Planned Behavior

**DOI:** 10.3390/pharmacy10060178

**Published:** 2022-12-19

**Authors:** Sharrel Pinto, Christopher Kotschevar, Aaron Hunt, Alex Middendorf, Christopher Robbins, Erin Miller, Deidra Van Gilder

**Affiliations:** 1 College of Pharmacy and Allied Health Professions, South Dakota State University, Brookings, SD 57007, USA; 2 Community Practice Innovation Center (CPIC), South Dakota State University, Brookings, SD 57007, USA; 3 Department of Allied and Population Health, College of Pharmacy and Allied Health Professions, South Dakota State University, Brookings, SD 57007, USA; 4 Department of Pharmacy Practice, College of Pharmacy and Allied Health Professions, South Dakota State University, Brookings, SD 57007, USA; 5 Pharmacy Quality Alliance, Alexandria, VA 22202, USA

**Keywords:** cardiovascular, community, diabetes, pharmacist, service

## Abstract

**Background**: Pharmacists can offer medication expertise to help better control diabetes and cardiovascular disease (CVD) and improve patient outcomes, particularly in rural communities. This project evaluated the impact of an awareness campaign on perceptions of expanded pharmacy services. **Methods**: The “Your Pharmacists Knows” campaign included a 30-s commercial, print material, and media announcements. A non-randomized pre-post study was completed using a modified theory of planned behavior (mTPB) to assess knowledge, attitude, perceived benefits and norms, and perceived control. A 73-item survey was administered to a convenience sample (*n* = 172) across South Dakota. Regression models to assess intent and utilization were conducted using age, gender, race, education, population, and insurance status as predictors for mTPB constructs. **Results**: Most common predictors were female gender and higher education level (*p* < 0.001). All mTPB constructs were significant predictors of intent to use services (*p* < 0.001). Knowledge and perceived control had the largest influence on intent. Additionally, there was significant improvement in post-campaign service utilization (*p* < 0.001). **Conclusions**: This campaign positively influenced intent to seek and utilize services in rural communities where pharmacies may be the only healthcare option for miles. Through targeted campaigns, patients with diabetes or CVD may find access to services to better manage their conditions.

## 1. Introduction

Patients in rural areas are known to experience inequities that contribute to healthcare disparities. Compared to urban areas, those in rural areas are more likely to have less education, reduced income, more children in poverty, and increased mortality [1]. South Dakota is one rural state with population characteristics that contribute to such healthcare disparities. The average population density of South Dakota is one of the lowest in the country at 11 people per square mile, far lower than the national average of 88.4 people per square mile [2]. Because of South Dakota’s rural nature, access to primary care providers (PCPs) and medical specialists is limited. Over three-quarters of South Dakota is categorized as a Health Profession Shortage Area and the same portion is considered a Medically Underserved Area/Population [3]. One survey of adults in South Dakota, Wyoming, North Dakota, and Montana reported that patients travel an average of 42 miles for a routine healthcare visit with some traveling more than 100 miles to see their PCPs [4].

Needing to travel long distances to reach a clinic poses a challenge for many rural patients. Community pharmacies are a uniquely positioned resource since, among other benefits, they are more accessible to many patients. It is reported that 64% of those living in South Dakota are located within 15 min of a pharmacy and 81% are located within 30 min [5]. Pharmacists have historically been underutilized as medication experts [6]. They are highly skilled and trained to improve care through medication therapy management (MTM), disease state education, improved medication adherence, cost-lowering strategies, and immunizations [7].

Such pharmacist-provided services are also positioned to help patients with diabetes, which is a major need throughout the United States, including in South Dakota. In 2018, 10.5% of the US population had a current diabetes diagnosis and 9.2% of the South Dakota population had a current diabetes diagnosis [8,9]. Each year, 1.5 million Americans are newly diagnosed with diabetes and in 2019, 26.8 of every 100,000 deaths in South Dakota were attributed to diabetes, higher than the national average of 21.6 per 100,000 deaths [8,9,10,11]. Diabetes is also linked to cardiovascular disease complications and patients with diabetes are two to four times more likely to die from heart diseases than those without diabetes [12]. The mortality implications of diabetes and cardiovascular disease in South Dakota is evident, with heart disease, stroke, and diabetes being three of the top ten leading causes of death in 2017 and poses a significant public health problem [13].

Despite available resources offered by pharmacists, it is estimated that thousands of South Dakotans have diabetes but are undiagnosed, and pharmacists remain underutilized as a resource to improve diabetes screening and care for South Dakotans [3]. In addition to undiagnosed diabetes, an estimated 20,000 South Dakotans have prediabetes [3]. To prevent and manage diabetes, heart disease, and stroke by implementing and evaluating evidence-based strategies to manage diabetes and CVD, as well as prevent or delay onset in high-burden populations, the Centers for Disease Control and Prevention (CDC) released a call to action [3]. In response to this call to action, the authors from South Dakota State University partnered with the South Dakota Department of Health on a 5-year project, through a cooperative agreement. This five-year project was designed to identify barriers faced by patients and develop programs to improve the care of South Dakotans, focusing on expanded pharmacy services [14]. In the first year of the project, a landscape analysis was completed of patients with diabetes, heart disease, and stroke. One major finding from this landscape analysis was that patients are unaware of the pharmacy services available, including MTM. Once these services were explained more in-depth, however, there was a consensus from patients that MTM and similar expanded services would be beneficial to their care [15]. Strand et al. observed that pharmacists are found in most of our local communities and often have more interaction with patients than their primary care providers and can therefore play a key role in reducing or helping patients manage these major public health concerns [16].

While pharmacists are present in most of our communities, there is currently insufficient data available on optimal ways to increase patient knowledge of pharmacy services in South Dakota. Thus, a need to improve awareness of various pharmacy-based services offered across the state was identified. Although awareness is directly linked to knowledge, it is important to also consider patients’ perspectives regarding pharmacy-based services. The Theory of Planned Behavior (TPB) is a theoretical framework that has previously been used to predict and describe health behaviors and intentions [17]. The TPB was first proposed in 1985 and has since been used to help motivate changes in public health areas such as smoking and alcohol cessation [18,19,20]. This theory has also been applied to motivating behavioral changes in patients diagnosed with diabetes and cardiovascular disease [21,22,23]. The TPB is unique because it takes into consideration personal intent. Intention is a central determinant of action and is impacted by attitude, perceived behavioral control, and social norms [24]. The TPB has previously demonstrated success in predicting health behaviors and offers great utility in public health projects [7].

As a result, the TPB was deemed an appropriate guide to understand patient perceptions of utilizing pharmacy services that are provided in community settings. The overall goal of this project was to understand the impact of a public health awareness campaign on the perceptions of expanded pharmacy services offered in South Dakota among people with diabetes and cardiovascular disease.

## 2. Methods

### 2.1. Study Design

The study design for this project was a non-randomized pre and post design using a survey that was administered through an electronic approach, using the online tool QuestionPro, and mail delivery in South Dakota from January 2020 to February 2022. All project procedures involving human subjects were approved by the South Dakota State University Institutional Review Board (1901020-EXM). An informed consent was required for each participant prior to recruitment in this project.

### 2.2. Conceptual Framework

A modified TPB (mTPB) was used to identify the salient constructs associated with pharmacy services, including MTM, Medication Therapy Review (MTR), diabetes education, cost-reduction, and medication adherence [18]. In conjunction with the mTPB, this project focused on factors that may influence participants’ motivation to get access to pharmacy services in the community setting. The TPB delineates theoretical constructs concerned with individual motivational factors as determinants of the likelihood of performing specific behaviors [18]. The three key constructs based on the TPB include (1) attitude, (2) perceived benefit and norms, and (3) perceived behavioral control. Attitude denotes an individual’s evaluation of gaining access to pharmacy services. For this project it was determined that a focus on perceived benefits versus the traditional construct of subjective norms, such as social pressure, would be more appropriate in assessing intent to utilize expanded pharmacy services. Perceived benefits and norms, then, are an individual’s perception about the use of pharmacy services by other members of the community. The perceived control represents an individual’s perceived ease or difficulty of approaching pharmacy services. While it is well recognized that these three constructs influence behavior, results from the landscape analysis in the first year of the CDC-funded project identified that a knowledge gap likely existed for patients in South Dakota. Thus, an assessment of knowledge was included in the survey design in addition to the three areas of the TPB.

### 2.3. Data Source

Recruitment of participants took place from October to December 2019 via newspaper and social media advertisements and flyers posted in community pharmacies across South Dakota. From the recruitment materials, potential participants called a central line to be screened for their eligibility in the survey. Screening was conducted by a research assistant who was not involved with the analysis of survey data collected. Inclusion criteria were individuals at least 18 years of age who self-reported a diagnosis of diabetes and/or cardiovascular disease. Individuals who were unable to complete a survey either electronically or via postal mail were excluded.

### 2.4. Awareness Campaign: “Your Pharmacist Knows”

An awareness campaign titled “Your Pharmacist Knows” was developed in Fall 2019. The campaign consisted of a 30-s commercial and print material, such as a poster, brochure, and business card. Additionally, the website Yourpharmacistknows.sdstate.edu housed all the information found on the print material and the commercial. The goal with these tools was to educate patients with diabetes and CVD on what pharmacists can do to better help their care. Topics covered included adherence tools, medication therapy management (MTM)/medication therapy review (MTR), immunizations, patient care and education, and cost-lowering measures.

Between Fall 2020 and Spring 2022, the poster was sent out to every pharmacy in South Dakota with the intention that it be displayed in the pharmacy to prompt patients to ask questions about the program and opportunities for them through the pharmacy. Pharmacies were asked to hang it on a visible bulletin board or near the register of the pharmacy or have it on counters for patients to view. The brochure was an alternative to the website. Pharmacies were asked to give these to patients who would like more information but may not be able to access the website due to limited internet access. The business cards were given to patients as a reminder to visit the website once they were home. Additionally, these materials were shared via social media across pharmacy organizations and the Department of Health social media pages. The commercial aired on various news stations and was streamed in 2-month blocks in Fall 2020, Spring 2021, Fall 2021, and Spring 2022.

### 2.5. Sample and Procedure

A convenience sampling technique was applied for participant recruitment. In January of 2020, the survey was distributed to eligible participants using the participants’ preferred method of postal mail or QuestionPro electronic distribution. The surveys were to be completed and returned to the project team within three weeks of distribution. This timeframe was later extended to 10 weeks to allow for an adequate response rate. Each returned survey was deidentified and assigned a unique identification number by a team member who was not associated with data analysis to maintain blinding to the analyzing project members. The nine-page survey was designed to take participants 20–30 min to complete. After each cycle of the awareness campaign enrolled participants were invited to complete the posttest survey. An example of the survey used can be found in Appendix A.

### 2.6. Measurements

To evaluate participants’ perceptions of pharmacy services provided in the community pharmacy, a 73-item survey was developed based on literature review and prior project findings [15,25,26,27,28]. The survey encompassed four constructs to measure participants’ perceptions of expanded pharmacy services including knowledge (16 items), attitude (14 items), perceived benefits and norms (14 items), and perceived behavioral control (14 items). The scores for each construct were calculated as a simple sum of responses. Additionally, sociodemographic and medication information was collected (13 items). The expanded pharmacy services measured in each section covered medication adherence, MTM, MTR, diabetes education, and cost-reduction. In addition, two items measured participants’ past use and their intent to use pharmacy services.

To ensure face and content validity, an expert panel consisting of seven pharmacy practitioners and educators examined the appropriateness of each survey item and resolved by consensus any issue encountered during the project [29]. Two different scoring systems were utilized to evaluate participants’ perceptions of expanded pharmacy services. In the knowledge section, a 3-point Likert-type scale (“True,” “False,” and “I don’t know”) was used to assess participant knowledge of pharmacy services. Correct marks were given a score of 1 and incorrect or “I don’t know” marks were scored as 0. The possible sum of knowledge scores ranged from 0 to 16; a higher score reflected greater awareness of pharmacy services provided in the community setting.

In the sections of attitude, perceived benefits and norms, and perceived behavioral control, each item was measured using a 5-point Likert-type scale with “strongly disagree” (score = 1) to “strongly agree” (score = 5) response options. The possible sum of scores for each subscale ranged from 14 to 70, with a higher score meaning a stronger attitude and perceptions of benefits and norms and behavioral control regarding the pharmacy services. Questions where a “strongly disagree” answer would be expected were scored inversely.

### 2.7. Statistical Analysis

Descriptive statistics, including mean, standard deviation (SD), and frequency were used to summarize the characteristics of the project samples. All continuous variables were expressed as the mean and SD and the categorical variables were expressed as numbers and percentages. The four primary outcomes included scores from each construct to include knowledge, attitude, perceived benefits and norms, and perceived behavior control regarding pharmacy services. Specific analyses conducted include independent samples *t*-tests to compare pre and post scores, chi-square analysis to compare group differences in both use of services and intent to use pharmacy services. Four separate multivariable linear regression analyses were performed to explore the association of each outcome variable with the six identified covariates based on literature review. Covariates used within the linear regression included age, gender, race, education, surrounding population density, and whether having health insurance impacted participants’ answers. The nominal covariates were categorized into dichotomous variables to minimize the risk of losing statistical power. Race was categorized as white and non-white and the highest education level was divided into two groups by using attainment of a high school diploma as the cutoff point. Surrounding population density was categorized in two groups depending on whether the geographic living area had more than 50,000 people, as reported by the participant. Health insurance status was divided into two groups: participants who self-reported having any type of health insurance and participants who self-reported not having health insurance.

Logistic regression was conducted to evaluate the impact of the mTPB constructs on intent to use services with a final logistic regression conducted to evaluate intent to use services as a predictor of services used. All statistical analyses were carried out using SPSS version 27.0 (IBM Corp; Armonk, NY), with the statistical significance level at a two-sided *p*-value < 0.05. To ensure adequate statistical power, a priori power analysis for a linear multiple regression with six predictors was performed using G*Power 3.1 with power set at 0.80 and two-tailed α = 0.05 [30]. As a result, a sample of at least 98 participants were required to reach a medium-sized effect (f^2^ = 0.15).

## 3. Results

### 3.1. Background Characteristics of the Participants

Of the 215 pre-surveys distributed, a total of 172 individuals responded, with a corresponding response rate at baseline of 80%. Forty-three respondents completed the follow-up survey resulting in a 25% response rate from baseline. Table 1 describes the participants’ demographic characteristics. The mean age of pre-survey participants was 62.9 years (SD = 11.6) compared to the post-survey participants with a mean age of 63.2 (SD = 12.9). Of the participants, 86 (50.0%) identified as female, 154 (89.5%) identified as non-Hispanic white, and 107 (62.2%) obtained at least an associate degree or higher. Around 70% of the participants (*n* = 113) lived in an area with a population greater than 50,000. The majority also self-reported having health insurance (*n* = 145, 89.5%). Hypertension, diabetes, and hypercholesterolemia were the three leading chronic illnesses that participants reported they had.

### 3.2. Self-Reported Medication Use of the Participants

Table 2 describes the results of participants’ self-reported medication-taking behaviors. Seventy-six (44.2%) participants said they always take their medications as prescribed. Approximately one-third of participants (*n* = 67) reported missing medication refills at least once throughout the year. The main reasons for missing refills included the cost of the medications (*n* = 24, 14.0%) and needing to see a doctor before obtaining refills (*n* = 20, 11.6%). Regarding medication-taking, 57 (34.5%) participants indicated that they sometimes missed taking medications. The most common cause for missing medication-taking was forgetfulness (*n* = 105, 61.0%).

### 3.3. Evaluation of Participants’ Perception and Intention of Pharmacy Services for Diabetes and Cardiovascular Disease

Table 3 shows the participants’ perception, in terms of knowledge, attitude, perceived benefits and norms, and perceived control, of pharmacy services for diabetes and cardiovascular disease. The average score of the participants’ knowledge of pharmacy services was 4.5 ± 2.9. The average score of the participants’ attitude of function, perceived benefits and norms, and perceived control of the use of pharmacy services was 45.3 ± 5.1, 43.2 ± 4.5, and 43.1 ± 3.8, respectively. The measures of participants’ knowledge, attitude, perceived benefits and norms, and perceived control yielded reliability coefficients with Cronbach’s alpha values of 0.81, 0.69, 0.61, and 0.76, respectively and all survey questions were retained. Additionally, there was significant improvement in all constructs over time (*p* < 0.001).

As shown in Table 4, only 15 participants (8.7%) indicated that they used pharmacy services over the past three months and only nine (5.6%) intended to use pharmacy services in the next three months. Of the 15 participants who had used pharmacy services in the past three months, nine reported that they had used a medication synchronization program and four said that they had attended heart disease education classes provided by pharmacists. Furthermore, nine patients reported that they would use a medication synchronization program, and five said that they would utilize a diabetes education class provided by pharmacists in the upcoming three months. Respondents that completed the post survey all indicated a significant increase in intent to utilize pharmacy services (*p* < 0.001). Post survey respondents indicated a significant increase in utilization of services (*p* < 0.05) with the exception being heart disease education classes (*p* = 0.345), however those utilizing heart disease education classes increased from 2.3% to 4.7%.

### 3.4. Association of Sociodemographic Characteristics with Perception of Pharmacy Services for Diabetes and Cardiovascular Disease

Table 5 presents the regression analyses of the association of sociodemographic characteristics with participant perception of pharmacy services. Participant knowledge was significantly impacted by three variables. Participants under 65 years old (*p* = 0.021), female participants (*p* < 0.001), or having an education degree more than high school level (*p* = 0.002) were associated with more knowledge of pharmacy services after controlling for the covariates (R^2^ = 0.256, *p* < 0.001). Of these three characteristics, the standardized coefficient indicated that female gender had the highest impact on knowledge, followed by education level, and finally age. Regarding attitude, female participants (*p* < 0.001), having an education degree more than high school level (*p* < 0.001), living area with less than 50,000 people (*p* = 0.023), or having health insurance (*p* = 0.003) were positively associated with participants’ attitudes of the function of pharmacy services (R^2^ = 0.354, *p* < 0.001). Of the characteristics demonstrating significant difference for attitude, higher education level had the most impact on attitude, followed by female gender, having health insurance, and living in an area with less than 50,000 people. For perceived benefits and norms, female participants (*p* < 0.001), non-white population (*p* < 0.001), or having an education degree more than high school level (*p* = 0.003) were more likely to report higher perceived norms of benefit from pharmacy services (R^2^ = 0.246, *p* < 0.001). Of these three characteristics, being non-white had the largest impact on perceived benefit and norms, followed by female gender, and higher educational level. Finally, after controlling for the other variables, having health insurance (*p* = 0.014) was the only determinant that was significantly associated with a higher perceived control of the use of pharmacy service (R^2^ = 0.072, *p* < 0.008).

### 3.5. TPB Framework and the Association of Constructs with Intent and Utilization of Services

**Figure 1:**
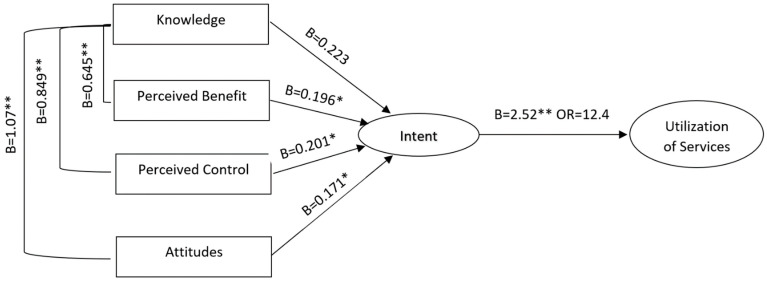
TPB constructs modeled on intent and utilization. B = Unstandardized Regression Estimate, OR = Odds Ratio, *p < 0.05, **p < 0.001.

Logistic regression modeling indicated a significant association for all mTPB constructs on participants intent to utilize pharmacy services related to diabetes and CVD. Figure 1 shows the predictive relationship of each construct on intent within the final model. Initial Knowledge was the most significant predictor followed by control, perceived benefit, and attitudes. All constructs had a significant association with intent at *p* < 0.05 with intent itself being a significant predictor of participants’ utilization of services (OR = 12.52, *p* < 0.001, 95% CI [3.5, 43.5]).

## 4. Discussion

There is a fairly broad body of literature examining factors that affect pharmacists’ intention to utilize drug monitoring programs, engage in MTM therapy, or other pharmaceutical care services [31,32,33,34]. However, using the TPB as a model for patient perceptions of pharmacy services is an understudied area, particularly in regard to rural populations [35]. A limited number of studies that have been conducted looking at patients’ intentions to use expanded pharmacy services through the lens of the TPB exist. There are two related studies conducted in Malaysia that assessed patients’ intention to utilize newly implemented pharmacy services that found a significant impact of attitude, social norms, and perceived control on intention to utilize services [36,37]. Additionally, one study that examined a focused New York City population of South Asian individuals found that attitudes and perceived control were significant predictors of intention to access pharmacy services [38].

We understand ours to be the first study to examine a rural population with the goal of understanding patient intentions to utilize pharmacy services related to diabetes and CVD. Ultimately, our work indicates the usefulness of using the TPB model to explore factors associated with patient perceptions of pharmacy services in rural areas. For our project, initial indication of lower baseline knowledge of pharmacy services came from focus groups and elicitation interviews in year one of the CDC 1815 project [16]. Information collected showed it was valuable to add a knowledge component to the TPB model. Since knowledge is shown to be an underlying component of intent, efforts to improve patient knowledge could be used to improve all three components of the TPB, thus improving intent to utilize enhanced pharmacy services.

The application of this model to rural communities of South Dakota contributes another layer of uniqueness to our project. By gathering rural patients’ perspective of expanding pharmacy services through the lens of the mTPB, a future educational campaign can be tailored to improve patient knowledge, attitude, perceived benefits and norms, and perceived behavior control of these services. Thus, a deeper dive into our findings, including those related to knowledge, attitude, perceived benefits and norms, and perceived behavior control, as well as how each of those factors are impacted by patient gender, education, insurance status, and race, will help unfold areas for future education for this rural population.

Patient knowledge regarding expanded pharmacy services was the strongest predictor of intent within our model. Initial patient knowledge was heavily influenced by patient demographics with demographic variables accounting for 25.6% of the adjusted variance. It is not surprising that pre-existing knowledge or familiarity with expanded pharmacy services was the most powerful predictor of intent. What we can learn from this, however, is that those with the most knowledge regarding pharmacy services were generally female, of younger age, and educated. This finding is not surprising since females are typically care givers and may have the tendency to frequent pharmacies more often as opposed to their male counterparts. Studies have shown that females in general utilize more prescription medication than males so it may be possible that females spend more time in pharmacies than men out of necessity [39,40]. While it is encouraging that younger adults and those with education had better knowledge of these services, patients often needing care and management for conditions such as diabetes and CVD tend to be older in age and those that have a lower educational status. This potentially indicates gaps within marketing or awareness strategies for these services and that a significant portion of pharmacy consumers are not being reached directly.

Patients’ pre-conceived attitudes had a similar predictive effect as knowledge and was the second most influential construct on intent and consistent with observations from other studies using the TPB [41]. Demographic variables accounted for the most predictive power of attitude out of all the mTPB constructs with demographics accounting for 35.4% of the variance. As with knowledge, the primary predictors were female gender and education, as well as health insurance. Since the construct of preconceived attitudes was related to patients’ perceptions of the relevance of the services and confidence in their pharmacist’s ability to deliver the services, this can provide useful information when marketing these services to the public. For example, over 68.4% of patients disagreed with the statement that it was easy for them to find ways to lower medication costs and 42.4% disagreed with the statement that medication cost saving tools were available. Patients were largely supportive of the ability of pharmacists to deliver disease specific care but seemed unsure of economic services that pharmacists could assist with. These types of economic factors can be important when patients are older and may have more medication needs or be uninsured or underinsured, one of the key components of social determinants of health. For example, a third of the patients in this project missed receiving their refills due to costs or going back to see a doctor to get a refill. Pharmacists can play a significant role by working with the patients’ physician to recommend cost-effective options for patients. Additionally, they can seek cost-saving options for medications that are expensive. Patients unaware of these services offered by pharmacists may fail to seek out pharmacists in time of need. Interestingly, our findings echoed comments made by elicitation interview and focus group participants who shared that as a result of being unable to fill their high-cost medications, they had to bear serious consequences, such as a foot amputation or being hospitalized. Adding more direct messaging across pharmacies and increasing awareness around how pharmacists can work with the patients’ physicians to help them receive more cost-effective care will be critical.

Perceived benefits and norms had a significant influence on patients’ intent to seek services. The perceived benefits and norms questions assessed patient’s beliefs in the benefits of expanded pharmacy practice services and whether they believed patients were utilizing them. Respondents predominately reported a “neutral” rating in over 70% of questions related to perceived benefits and norms. This indicates that participants need additional messaging regarding the benefits of pharmacy services particularly related to medication therapy review and the benefits of having a 90-day supply of medication as opposed to a 30-day supply. While most of our patients came from more populated areas in the state, the 90-day refill model would certainly help patients and families living in more rural parts of the state. Studies have found multiple trips to the pharmacy can lead to medication non-adherence due to gaps in fill dates [42]. Within perceived benefit, race had the largest impact, with non-white individuals perceiving better benefits surrounding pharmacy services. Interestingly, race demonstrated significance only in regard to perceived benefit. South Dakota is home to a large population of Native Americans, many of whom are served by the United States Indian Health Service (IHS). The IHS has long been known for holistic pharmacy services provided to patients [43,44,45]. IHS pharmacists have established standards of pharmacy practice by accessing patients’ health record, immunization status, and past medical history to assess the appropriateness of drug therapy and manage medication therapy and disease. This could explain the difference in the perceived benefits that differ between communities. For those that use the IHS system for their healthcare, pharmacists providing expanded services may be more commonplace than in other areas of South Dakota.

Perceived control had the lowest impact on patients’ intent to use services, though it was still a significant predictor. Perceived control measured the patient’s confidence that they could engage with the pharmacist and participate in the services offered at a pharmacy. Like with perceived benefits, patients had neutral responses to 65% of questions indicating that some patients may not have had a high level of confidence in the ease of accessing services. Patients did have a high level of agreement related to participating in and requesting MTM services, medication synchronization, and ease of speaking with their pharmacist about diabetes. Demographic factors had the smallest effect on perceived control in all the models with demographics only accounting for 7.2% of the variance. Insurance status was the only demographic characteristic that showed significant impact on perceived control among the independent variables tested. Whether this is due to those who are insured being of higher socioeconomic status, having a better grasp on their healthcare in general, or for other reasons is unclear and calls for further consideration.

Our findings prove beneficial for developing ways to better advertise pharmacy services to patients and educate patients on the benefits of these services to promote overall public health. Wood et al. highlighted the struggle of creating buy-in from patients regarding pharmacy services, including MTM and diabetes education services [46]. In their study, despite a plethora of advertising techniques including radio advertisement and personal selling of services to patients, less than 10% of patients enrolled in the offered services. To help avoid similar results, our theory-based approach to patient baseline awareness will help inform future development of educational intervention.

The timeline for our project spans both pre-COVID and post-COVID periods, which introduced challenges. While there certainly has been recognition of the role of the pharmacist in public health emergency situations, there still seems to be hesitation across states on provider status and lack of awareness of the key roles pharmacists continue to play regarding public health. In rural and frontier states such as South Dakota where pharmacists do not currently have provider status but are almost always one of the most accessible healthcare workers, it is imperative that communities utilize pharmacists for prevention and management, especially as it relates to chronic conditions. Studies consistently show that pharmacists can have a significant impact in helping patients minimize their healthcare costs [47,48,49]. However, if patients do not recognize pharmacists as a resource or are not reaching out, healthcare costs across rural and underserved communities may continue to be negatively impacted. Future campaign initiatives would benefit from focusing on specific tools utilized by pharmacists or examples of how pharmacists can assist patients in these cost-saving measures.

From our results, we suggest addressing four specific areas to influence patient intention and behavior. By influencing patient knowledge, attitude, perceived benefits and norms, and perceived behavior control as it relates to pharmacy services, more successful education and awareness of patients should occur, whether that be on an individual level or for populations across rural and urban communities. To have the best potential impacts, since patient gender and education level impacted both knowledge and attitude, which were found to be the two biggest factors of the modified TPB, a focus for future awareness campaigns in South Dakota should be tailored more towards male patients and patients with lower education.

### Strengths and Limitations

Strengths of this project include that the survey was theory driven, being rooted in the TPB. The extensiveness of this survey and a high initial patient participation rate also lend to its strengths. Additionally, regression data analysis that was controlled for covariates allowed for an in depth look at which variables influenced each of the three constructs of the TPB in addition to which variables influenced patient knowledge of expanded pharmacy services.

Limitations to this project include the low post survey response rate. It is possible that the length of the survey contributed to participant fatigue. Additionally, with the sample being a convenience sample, bias likely exists among the survey non-respondents. It is possible that participants who did not respond to the post survey may have had different perceptions that those found through our findings. Finally, the self-reported design of both our inclusion criteria and our survey response could have led to biases, such as social desirability, in patient reporting of certain data. Over fifty percent of respondents were from non-rural areas, future studies should attempt to capture more rural respondents as there is still a dearth of information regarding pharmacy access (pharmacy deserts) for truly rural inhabitants.

## 5. Conclusions

Rural and underserved communities can have public health allies in pharmacists and pharmacies. Through public health awareness campaigns such as “Your Pharmacist Knows,” pharmacists in states such as South Dakota may help minimize the healthcare burden of rural and frontier communities. This project demonstrated that even with a modest awareness campaign launched twice a year, participants’ intention and usage of pharmacy resources can be increased, which can subsequently translate to positive public health change. Additionally, while the campaign impacted females, younger adults, those with higher education and insurance coverage, there is room to expand to cover those that come from lower socio-economic backgrounds that would benefit from pharmacy services.

## Figures and Tables

**Table 1:** Description of participant demographics (Pre-Survey *n* = 172, Post-Survey *n* = 43).

	Pre-Survey		Post Survey	
Variables	*n* (%)	mean (SD)	*n* (%)	mean (SD)
*Sociodemographic background*				
**Age (in years):** range 19–88		62.9 (11.6)		63.2 (12.9)
**Gender**				
Female	86 (50.0)		25 (58.1)	
Male	86 (50.0)		18 (41.9)	
**Race**				
Non-Hispanic white	154 (89.5)		39 (90.7)	
Black	0 (0.0)		0 (0.0)	
American Indian/Alaska Native	2 (1.2)		2 (4.7)	
Multiple Race	13 (7.6)		1 (2.3)	
Not Reported	3 (1.7)		1 (2.3)	
**Education**				
High school or less	18 (10.5)		8 (18.6)	
High school graduate or GED	47 (27.3)		9 (20.9)	
Associate’s degree or a 2-year college degree	47 (27.3)		12 (27.9)	
Bachelor’s degree or a 4-year college degree	38 (22.1)		7 (16.3)	
Master’s or doctoral degree	22 (12.8)		7 (16.3)	
**Population of living surroundings**				
Less than 1000	2 (1.2)		11 (25.6)	
1001–5000	9 (5.2)		9 (20.9)	
5001–20,000	32 (18.6)		10 (23.3)	
20,001–50,000	16 (9.3)		2 (4.6)	
More than 50,000	113 (65.7)		11 (25.6)	
**Health insurance**				
Private	80 (46.5)		13 (30.2)	
Medicaid	14 (8.1)		0 (0.0)	
Medicare	18 (10.5)		11 (25.6)	
Other	0 (0.0)		3 (7.0)	
Multiple	42 (24.4)		12 (27.9)	
Uninsured	18 (10.5)		4 (9.3)	
*Clinical characteristics*				
**Chronic illnesses**				
Hypertension	94 (43.5)		29 (69.4)	
Diabetes	83 (48.3)		25 (52.8)	
Hypercholesterolemia	47 (27.3)		20 (41.7)	
Heart attack	17 (10.5)		6 (16.7)	
Stroke	17 (10.5)		2 (5.5)	
Peripheral vascular disease	21 (13.0)		2 (5.5)	

**Table 2:** Description of participant behaviors of medication use (Pre-Survey *n* = 172, Post-Survey *n* = 43).

Variables	*n* (%) Pre-Survey	*n* (%) Post- Survey
Behaviors of medication use		
Take the medication per direction		
Almost never	0 (0.0)	1 (2.3)
Sometimes	31 (18.0)	1 (2.3)
Almost always	65 (37.8)	9 (20.9)
Always	76 (44.2)	32 (74.4)
Frequency of missing medication refill		
Never	105 (61.0)	30 (69.8)
1–2 times per year	42 (24.4)	9 (20.9)
3–4 times per year	13 (7.6)	0 (0)
5–6 times per year	0 (0.0)	4 (9.3)
Currently don’t take any medications	12 (7.0)	0 (0.0)
Reasons of missing medication refill		
I don’t forget to refill my prescriptions	115 (66.9)	23 (53.5)
Need to see a doctor before refill	20 (11.6)	9 (20.9)
Cost	24 (14.0)	2 (4.7)
Cannot get to the pharmacy	12 (7.0)	5 (11.6)
Other	1 (0.6)	1 (2.3)
Frequency of missing medication-taking		
Never	54 (32.7)	12 (31.6)
Almost never	54 (32.7)	22 (57.9)
Sometimes	57 (34.5)	3 (7.9)
Most of the time	0 (0)	1 (2.6)
Reasons of missing medication-taking		
I don’t forget	59 (34.3)	11 (25.6)
Forgetfulness	105 (61.0)	23 (53.5)
Fear of side effects	56 (32.6)	3 (7.0)
Doubts of medication effects	24 (14.0)	2 (4.7)
Other	3 (1.7)	10 (23.3)

**Table 3:** Evaluation of participant perceptions of pharmacy services for diabetes and cardiovascular disease (Pre- Survey *n* = 172, Post-Survey *n* = 43).

	Pre-Survey		Post Survey		
Variables	Mean (SD) ^a^	Range	Mean (SD) ^a^	Range	*p* Value
Awareness of pharmacy services (full score = 16)	4.5 (2.9)	0–11	9.0 (3.6)	1–16	<0.001
Attitude of function of pharmacy services (full score = 70)	45.3 (5.1)	36–63	52.5 (7.4)	34–70	<0.001
Norms of benefit from pharmacy services (full score = 70)	43.2 (4.5)	35–57	49.5 (7.2)	33–68	<0.001
Perceived control of the use of pharmacy services (full score = 70)	43.1 (3.8)	36–55	49.3 (7.9)	25–67	<0.001

^a^: SD = Standard Deviation.

**Table 4:** Description of participant intentions and experiences of using pharmacy services (Pre- Survey *n* = 172, Post-Survey *n* = 43).

Variables	*n* (%) Pre-Survey	*n* (%) Post-Survey	*p* Value
Experiences of using pharmacy services over the past three months			
Medication therapy management	0 (0.0)	4 (9.3)	<0.001
Medication therapy review	1 (0.6)	6 (14.0)	<0.001
Medication synchronization program	9 (5.2)	9 (20.9)	0.003
Diabetes education classes provided by pharmacists	1 (0.6)	3 (7.0)	0.026
Heart disease education classes provided by pharmacists	4 (2.3)	2 (4.7)	0.345
Intention to use pharmacy services in the next three months			
Medication therapy management	3 (1.7)	11 (25.6)	<0.001
Medication therapy review	4 (2.3)	9 (20.9)	<0.001
Medication synchronization program	9 (5.2)	15 (34.9)	<0.001
Diabetes education classes provided by pharmacists	5 (2.9)	10 (23.3)	<0.001
Heart disease education classes provided by pharmacists	3 (1.7)	10 (23.3)	<0.001

**Table 5:** Multiple linear regression: Knowledge, attitude, social norms, and perceived behavior control regressed on demographics (*n* = 172).

		Attitude			Perceived Benefits			Perceived Control			Knowledge	
Independent Variable	B	SE	*p* value	B	SE	*p* value	B	SE	*p* value	B	SE	*p* value
Age	−0.03	0.029	0.291	0.026	0.069	0.334	−0.008	0.025	0.737	−0.042	0.018	0.021
Female	3.978	0.664	<0.001	2.313	0.599	<0.001	1.014	0.556	0.07	2.013	0.401	<0.001
White ^a^	−0.068	1.121	0.951	−5.297	1.113	<0.001	0.181	1.021	0.86	1.35	0.744	0.072
Highest education degree more than high school level ^b^	5.075	0.735	<0.001	2.009	0.673	0.003	1.217	0.618	0.051	1.445	0.45	0.002
Living area with more than 50,000 people	−1.847	0.803	0.023	0.411	0.702	0.559	0.494	0.652	0.45	0.831	0.469	0.078
Health insurance	2.991	0.983	0.003	1.813	0.966	0.062	2.197	0.083	0.014	1.054	0.646	0.105
*F*	13.947			9.723			3.030			10.173		
*Adjusted R*^2^	0.354			0.246			0.072			0.256		

Note: B: unstandardized coefficient, SE: standard error. ^a^ Compared with non-white participants; ^b^ Compared with those with highest education degree less than some college or technical school.

## Data Availability

Not applicable.

## Appendix A. Pharmacy Expanded Services Survey

Please answer the following questions by filling in the bubble with your answer to each question. Return your completed survey to the study team using the included envelope. Please do not place your name on the form. All your responses are confidential.

Section 1—The following statements are about your awareness of some pharmacy services for diabetes and heart disease. Please fill in a bubble for either True, False, or “I don’t know” for each of the following statements based on your awareness for each statement.

**Table A1:** Awareness of some pharmacy services for diabetes and heart disease.

	True	False	I Don’t Know
1. Pharmacists cannot participate in the National Diabetes Prevention Program.	○	○	○
2. I am aware of what the term “DSME” stands for when it comes to diabetes education.	○	○	○
3. I know what Medication Therapy Management (MTM) is.	○	○	○
4. I know what a Medication Sync program is.	○	○	○
5. A Medication Sync program does NOT help to schedule my medications to be filled by the pharmacy all at the same time.	○	○	○
6. Medication Therapy Management (MTM) is a group of services offered by pharmacists only at a doctor’s office.	○	○	○
7. A Medication Therapy Review (MTR) consists of more than assessing barriers to proper medication use.	○	○	○
8. Pharmacists can help me pick lower cost over the counter (OTC) medication products.	○	○	○
9. Some pharmacies offer diabetes education classes.	○	○	○
10. The National Diabetes Prevention Program focuses on healthy eating and physical activity.	○	○	○
11. Education from my pharmacist on how I could better manage diabetes or heart disease can help keep me out of the hospital.	○	○	○
12. Pharmacists can help with getting a 90-day supply of a prescription instead of a 30-day supply.	○	○	○
13. Medication Therapy Management (MTM) is a program where pharmacists make sure my medications are working and safe.	○	○	○
14. A pharmacist communicating with your doctor is an important part of Medication Therapy Review (MTR).	○	○	○
15. Medication discount cards are NOT available to help lower prescription drug costs.	○	○	○
16. Pharmacists are able to provide education classes about certain diseases.	○	○	○

Section 2—The following statements are regarding your attitude towards pharmacy services focused on diabetes and heart disease. There are no right or wrong answers. Please answer each statement based on how much you agree or disagree towards each statement.

**Table A2:** Attitude towards pharmacy services focused on diabetes and heart disease.

	Strongly Disagree	Disagree	Neutral	Agree	Strongly Agree
1. Having my pharmacist schedule my medications to be refilled at the same time will help me to fill my medications on time.	○	○	○	○	○
2. Having my pharmacist ask me about how I take my medications could be beneficial for me.	○	○	○	○	○
3. I am confident my pharmacist could perform a Medication Therapy Review (MTR) to make sure I am getting the right treatment.	○	○	○	○	○
4. I believe there are many cost savings tools for medications.	○	○	○	○	○
5. I would allow my pharmacist to teach me about the diseases I am using medications to treat.	○	○	○	○	○
6. I would want to participate in the National Diabetes Prevention Program that is provided by my pharmacist.	○	○	○	○	○
7. I would like if my pharmacist could provide education on how I can manage diabetes or heart disease better.	○	○	○	○	○
8. Having 90-day supply of medications from the pharmacy would help me miss fewer doses of medication.	○	○	○	○	○
9. Medication therapy management (MTM) should be offered at least once a year to me.	○	○	○	○	○
10. I think a Medication Therapy Review (MTR) should addresses several different things related to medications including potential barriers to health, medication-related problems, and proper medication use.	○	○	○	○	○
11. It is easy to find ways to lower costs for medications.	○	○	○	○	○
12. I believe my pharmacist can teach me about my diseases.	○	○	○	○	○
13. I feel comfortable with my pharmacist helping me with healthy eating and physical activity recommendations.	○	○	○	○	○
14. I do NOT want my pharmacist to provide more education on how I can manage diabetes or heart disease better.	○	○	○	○	○

Section 3—The following statements are regarding how some pharmacy services might impact patients taking medications for diabetes and heart disease. There are no right or wrong answers. Please answer each statement based on how much you agree or disagree towards each statement.

**Table A3:** How some pharmacy services might impact patients taking medications for diabetes and heart disease.

	Strongly Disagree	Disagree	Neutral	Agree	Strongly Agree
1. Patients with diabetes and heart disease would benefit from being in a Medication Sync program.	○	○	○	○	○
2. Patients with diabetes and heart disease would benefit from Medication Therapy Management (MTM) which could help them learn about their medications.	○	○	○	○	○
3. Patients with diabetes and heart disease would benefit from Medication Therapy Review (MTR), which could help find potential problems with their medications.	○	○	○	○	○
4. Patients with diabetes and heart disease know about the different ways to save money on medications.	○	○	○	○	○
5. Patients with diabetes and heart disease would benefit by having diabetes and heart disease education from their pharmacist.	○	○	○	○	○
6. Patients with diabetes and heart disease would have their pharmacist help them with healthy eating and physical activity.	○	○	○	○	○
7. Patients with diabetes and heart disease would have their pharmacist teach them how to better manage diabetes or heart disease.	○	○	○	○	○
8. It is more convenient for patients with diabetes and heart disease to get a 90-day supply of medications rather than a 30-day-supply of medications.	○	○	○	○	○
9. Medication Therapy Management (MTM) helps many patients with diabetes and heart disease by teaching them about medications and how to organize them, as well as any changes they need to make.	○	○	○	○	○
10. Patients with diabetes and heart disease are using Medication Therapy Review (MTR) sessions provided by pharmacists.	○	○	○	○	○
11. It is easy for patients with diabetes and heart disease to find ways to save money on medications.	○	○	○	○	○
12. People with diabetes benefit from using pharmacists as diabetes educators.	○	○	○	○	○
13. Patients with diabetes and heart disease would benefit from a pharmacist participating in the National Diabetes Prevention Program.	○	○	○	○	○
14. Patients with diabetes and heart disease would benefit from the pharmacist teaching them more about how to better manage their diabetes or heart disease.	○	○	○	○	○

Section 4—The following statements are regarding how easy it might be for you to use some pharmacy services for your medications and health. There are no right or wrong answers. Please answer each statement based on how much you agree or disagree towards each statement.

**Table A4:** How easy it might be for you to use some pharmacy services for your medications and health.

	Strongly Disagree	Disagree	Neutral	Agree	Strongly Agree
1. Enrollment in a program that would schedule all my medications to refill at the same time is up to me.	○	○	○	○	○
2. Participating in Medication Therapy Management (MTM) completely up to me.	○	○	○	○	○
3. I am able to ask my pharmacist to tell me about what Medication Therapy Review (MTR) involves.	○	○	○	○	○
4. I have control over what my medication costs are.	○	○	○	○	○
5. I am able to ask my pharmacist about diabetes education.	○	○	○	○	○
6. If my pharmacist participated in the National Diabetes Prevention Program, I am able to attend classes at the pharmacy.	○	○	○	○	○
7. I am able to talk with my pharmacist about diabetes and heart disease education.	○	○	○	○	○
8. I am able to request a 90-day supply of my medications from the pharmacy.	○	○	○	○	○
9. I am able to request a Medication Therapy Management (MTM) session with my pharmacist.	○	○	○	○	○
10. I am confident I could find out what a Medication Therapy Review (MTR) consists of by asking my pharmacist.	○	○	○	○	○
11. I can ask my pharmacist about ways to lower my medication costs.	○	○	○	○	○
12. I can ask my pharmacist questions about diabetes.	○	○	○	○	○
13. I can look for more information at my pharmacy about the National Diabetes Prevention Program.	○	○	○	○	○
14. I would NOT be able to visit with my pharmacist if I had questions about how I could better manage my diabetes or heart disease.	○	○	○	○	○

Section 5—The following questions are focused on if you have used any of the services talked about in this survey. Please answer them to the best of you ability.

**Table A5:** If you have used any of the services talked about in this survey.

1.	I intend to use the following services in the next three (3) months (select all that apply):	
	○	Medication Therapy Management (MTM) provided by my pharmacist.
	○	Medication Therapy Review (MTR) provided by my pharmacist.
	○	Medication Sync program
	○	Diabetes education classes provided by my pharmacist.
	○	Heart disease education provided by my pharmacist.
2.	I have used the following services in the past three (3) months (select all that apply):	
	○	Medication Therapy Management (MTM) provided by my pharmacist.
	○	Medication Therapy Review (MTR) provided by my pharmacist.
	○	Medication Sync program
	○	Diabetes education classes provided by my pharmacist.
	○	Heart disease education provided by my pharmacist.

Section 6—The following questions are used to help us know a little more about you but are not used to identify you in any way. Please answer the following questions.

**Table A6:** Participants Information.

5.	What is your age?	
	____________ years old	
6.	What is your gender?	
	○	Male
	○	Female
	○	Other
7.	Are you Hispanic or Latino?	
	○	Yes
	○	No
8.	How would you describe your race? Please check all that apply.	
	○	White
	○	Black or African-American
	○	American Indian or Alaska Native
	○	Asian
	○	Native Hawaiian or Pacific Islander
	○	Other, please specify: _________________________________________
9.	What is the highest level of education you have completed? Please choose only one.	
	○	Did not graduate high school
	○	High school graduate or GED
	○	Associate’s degree or a 2-year college degree
	○	Bachelor’s degree or a 4-year college degree
	○	Master’s or doctoral degree
10.	In the town or city that you live, what is the approximate population?	
	○	Less than 1,000
	○	1,001–5,000
	○	5,001–20,000
	○	20,001–50,000
	○	More than 50,000
11.	What type of medication insurance do you have (select all that apply)?	
	○	Private insurance (through an employer or other)
	○	Medicaid
	○	Medicare Part D
	○	Other
	○	I do not have insurance
12.	Which of the following conditions do you have? Select all that apply:	
	○	Diabetes
	○	High blood pressure
	○	High cholesterol
	○	I have had a heart attack
	○	I have had a stroke
	○	I have peripheral vascular disease
13.	How often do you follow the medication directions when taking medications?	
	○	Never
	○	Almost never
	○	Sometimes
	○	Almost always
	○	Always
14.	How often do you miss refilling your prescriptions?	
	○	Never
	○	1–2 times per year
	○	3–4 times per year
	○	5–6 times per year
	○	7 or more times per year
	○	I do not currently take any medications
15.	What are some reasons you forget to refill your prescription?	
	○	I don’t forget to refill my prescriptions
	○	Need to see a doctor before refilling
	○	Cost
	○	Cannot get to the pharmacy
	○	Other (please explain): _____________________________________________
16.	How often do you miss taking your medications?	
	○	Never
	○	Almost never
	○	Sometimes
	○	Almost always
	○	Always
17.	What are some reasons you miss taking your medications (select all that apply)?	
	○	I don’t miss taking my medications
	○	I forget
	○	There are side effects
	○	I don’t think the medication is working
	○	Other (please explain): _______________________________________________

At this time, please put this survey in the provided envelope and place it the mail. Thank you for participating in our survey. In the spring, another survey with the same questions will be sent out. Please fill out the survey again at that point. Following the return of the spring survey, the $50 gift card will be sent to you.

## References

[1] Cecil G., Sheps Center for Health Services Research Rural Health Snapshot 2017. Published 2017. https://www.shepscenter.unc.edu/wp-content/uploads/dlm_uploads/2017/05/Snapshot2017.pdf.

[2] US Census Bureau QuickFacts: South Dakota. Published 1 July 2021. https://www.census.gov/quickfacts/SD.

[3] South Dakota Department of Health South Dakota Diabetes State Plan 2018–2020. Published 2018. https://doh.sd.gov/prevention/assets/diabetesstateplan2018.pdf.

[4] Mattson J.W. (2011). Aging and Mobility in Rural and Small Urban Areas: A Survey of North Dakota. J. Appl. Gerontol..

[5] South Dakota Department of Health South Dakota’s Need for Medication Therapy Management: Diabetes Prevalence, Blood Pressure Medication Nonadherence, and Pharmacy Access. https://doh.sd.gov/documents/diseases/chronic/MTM_GIS_Document.pdf.

[6] Smith M.A., Spiggle S., McConnell B. (2017). Strategies for community-based medication management services in value-based health plans. Res. Soc. Adm. Pharm..

[7] Goode J.V., Owen J., Page A., Gatewood S. (2019). Community-Based Pharmacy Practice Innovation and the Role of the Community-Based Pharmacist Practitioner in the United States. Pharmacy.

[8] Centers for Disease Control and Prevention National Diabetes Statistics Report, 2020. Published 2020. https://www.cdc.gov/diabetes/data/statistics-report/index.html.

[9] American Diabetes Association The Burden of Diabetes in South Dakota. Published October 2021. https://diabetes.org/sites/default/files/2021-11/ADV_2021_State_Fact_sheets_South%20Dakota_rev.pdf.

[10] Centers for Disease Control and Prevention Stats of the States-Diabetes Mortality. Published 11 February 2021. https://www.cdc.gov/nchs/pressroom/sosmap/diabetes_mortality/diabetes.htm.

[11] Murphy S.L. (2021). Mortality in the United States, 2020. NCHS Data Brief.

[12] American Diabetes Association (2018). Economic Costs of Diabetes in the U.S. in 2017. Diabetes Care.

[13] Centers for Disease Control and Prevention Stats of the State of South Dakota. Published 2018. https://www.cdc.gov/nchs/pressroom/states/southdakota/southdakota.htm.

[14] Pinto S., Dickinson A., Middendorf A., Hawkins-Taylor C. (2019). Pharmacology Focus: Breaking Barriers to Improve Medication Management in Practice. S. Dak. Med. J. S. Dak. State Med. Assoc..

[15] South Dakota Department of Health Year 1: Landscape Analysis Final Report. Published 2019. https://doh.sd.gov/documents/diseases/chronic/MTM_SDSU_Year1_FinalReport_CDC.pdf.

[16] Strand M.A., DiPietro Mager N.A., Hall L., Martin S.L., Sarpong D.F. (2020). Pharmacy Contributions to Improved Population Health: Expanding the Public Health Roundtable. Prev. Chronic Dis..

[17] Young H.M., Lierman L., Powell-Cope G., Kasprzyk D., Benoliel J.Q. (1991). Operationalizing the theory of planned behavior. Res. Nurs. Health.

[18] Ajzen I., Kuhl J., Beckmann J. (1985). From Intentions to Actions: A Theory of Planned Behavior. Action Control.

[19] Bledsoe L.K. (2006). Smoking cessation: An application of theory of planned behavior to understanding progress through stages of change. Addict. Behav..

[20] Sharma M., Kanekar A. (2007). Theory of Reasoned Action & Theory of Planned Behavior in Alcohol and Drug Education. J. Alcohol. Drug Educ..

[21] White K.M., Terry D.J., Troup C., Rempel L.A., Norman P., Mummery K., Riley M., Posner N., Kenardy J. (2012). An extended theory of planned behavior intervention for older adults with type 2 diabetes and cardiovascular disease. J. Aging Phys. Act..

[22] Lee L.T., Bowen P.G., Mosley M.K., Turner C.C. (2017). Theory of Planned Behavior: Social Support and Diabetes Self-Management. J. Nurse Pract..

[23] Beiranvand S., Asadizaker M., Fayazi S., Yaralizadeh M. (2015). Efficacy of an Intervention Based on the Theory of Planned Behavior on Foot Care Performance in Type II Diabetic Patients. Jundishapur J. Chronic Dis. Care.

[24] Reid A.E., Aiken L.S. (2011). Integration of five health behaviour models: Common strengths and unique contributions to understanding condom use. Psychol. Health.

[25] Urmie J.M., Farris K.B., Herbert K.E. (2007). Pharmacy students’ knowledge of the Medicare drug benefit and intention to provide Medicare medication therapy management services. Am. J. Pharm. Educ..

[26] Tan C.L., Hassali M.A., Saleem F., Shafie A.A., Aljadhey H., Gan V.B. (2015). Development, test-retest reliability and validity of the Pharmacy Value-Added Services Questionnaire (PVASQ). Pharm. Pract..

[27] Mailloux L., Yates S., Spencer K., Davis J., Chen A.M.H., Franz T. (2018). Changing Patient Perceptions of MTM: Determining an Effective Method of Education. Innov. Pharm..

[28] Montaño D.E., Kasprzyk D. (2015). Theory of reasoned action, theory of planned behavior, and the integrated behavioral model. Health Behavior Theory, Research, and Practice.

[29] Haynes S.N., Richard D.C.S., Kubany E.S. (1995). Content validity in psychological assessment: A functional approach to concepts and methods. Psychol. Assess..

[30] Faul F., Erdfelder E., Buchner A., Lang A.G. (2009). Statistical power analyses using G*Power 3.1: Tests for correlation and regression analyses. Behav. Res. Methods.

[31] Fleming M.L., Bapat S.S., Varisco T.J. (2019). Using the theory of planned behavior to investigate community pharmacists’ beliefs regarding engaging patients about prescription drug misuse. Res. Social Adm. Pharm..

[32] Gavaza P., Fleming M., Barner J.C. (2014). Examination of psychosocial predictors of Virginia pharmacists’ intention to utilize a prescription drug monitoring program using the theory of planned behavior. Res. Social Adm. Pharm..

[33] Herbert K.E., Urmie J.M., Newland B.A., Farris K.B. (2006). Prediction of pharmacist intention to provide Medicare medication therapy management services using the theory of planned behavior. Res. Social Adm. Pharm..

[34] Rawy M., Look K.A., Amin M.E.K., Chewning B. (2021). Development and validation of a theory-based instrument to predict community pharmacists’ intention to provide pharmaceutical care services. Res. Social Adm. Pharm..

[35] Yuan C., Ding Y., Zhou K., Huang Y., Xi X. (2019). Clinical outcomes of community pharmacy services: A systematic review and meta-analysis. Health Soc. Care Community.

[36] Tan C.L., Hassali M.A., Saleem F., Shafie A.A., Aljadhay H., Gan V.B. (2016). Building intentions with the theory of planned behaviour: A qualitative assessment of salient beliefs about pharmacy value added services in Malaysia. Health Expect..

[37] Tan C.L., Gan V.B., Saleem F., Hassali M.A. (2016). Building intentions with the Theory of Planned Behaviour: The mediating role of knowledge and expectations in implementing new pharmaceutical services in Malaysia. Pharm. Pract..

[38] Hasan S.A., Muzumdar J.M., Nayak R., Wu W.K. (2019). Using the Theory of Planned Behavior to Understand Factors Influencing South Asian Consumers’ Intention to Seek Pharmacist-Provided Medication Therapy Management Services. Pharmacy.

[39] Orlando V., Mucherino S., Guarino I., Guerriero F., Trama U., Menditto E. (2020). Gender Differences in Medication Use: A Drug Utilization Study Based on Real World Data. Int. J. Environ. Res. Public Health.

[40] Manteuffel M., Williams S., Chen W., Verbrugge R.R., Pittman D.G., Steinkellner A. (2014). Influence of patient sex and gender on medication use, adherence, and prescribing alignment with guidelines. J. Womens Health.

[41] Ajzen I. (1991). The theory of planned behavior. Organ. Behav. Hum. Decis. Process..

[42] Meddings J., Kerr E.A., Heisler M., Hofer T.P. (2012). Physician assessments of medication adherence and decisions to intensify medications for patients with uncontrolled blood pressure: Still no better than a coin toss. BMC Health Serv. Res..

[43] Fisher R., Brands A., Herrier R. (1995). History of the Indian Health Service Model of Pharmacy Practice: Innovations in Pharmaceutical Care. Pharm. Hist..

[44] Duvivier H., Gustafson S., Greutman M., Jangchup T., Harden A.K., Reinhard A., Warshany K. (2017). Indian Health Service pharmacists engaged in opioid safety initiatives and expanding access to naloxone. J. Am. Pharm. Assoc..

[45] Berg S. How Embedded Pharmacists Improve Care for Native American patients. American Medical Association. Published 2020. https://www.ama-assn.org/practice-management/scope-practice/how-embedded-pharmacists-improve-care-native-american-patients.

[46] Wood K.D., Offenberger M., Mehta B.H., Rodis J.L. Community Pharmacy Marketing: Strategies for Success. Published online 2011. http://conservancy.umn.edu/handle/11299/116874.

[47] Dalton K., Byrne S. (2017). Role of the pharmacist in reducing healthcare costs: Current insights. Integr. Pharm. Res. Pract..

[48] Ni W., Colayco D., Hashimoto J., Komoto K., Gowda C., Wearda B., McCombs J. (2018). Reduction of healthcare costs through a transitions-of-care program. Am. J. Health-Syst. Pharm..

[49] Rajiah K., Sivarasa S., Maharajan M.K. (2021). Impact of Pharmacists’ Interventions and Patients’ Decision on Health Outcomes in Terms of Medication Adherence and Quality Use of Medicines among Patients Attending Community Pharmacies: A Systematic Review. Int. J. Env. Res. Public Health.

